# Tissue Engineering Approaches for Enamel, Dentin, and Pulp Regeneration: An Update

**DOI:** 10.1155/2020/5734539

**Published:** 2020-02-25

**Authors:** Geraldine M. Ahmed, Eman A. Abouauf, Nermeen AbuBakr, Christof E. Dörfer, Karim Fawzy El-Sayed

**Affiliations:** ^1^Stem Cell and Tissue Engineering Research Group, Faculty of Dentistry, Cairo University, Cairo, Egypt; ^2^Department of Endodontics, Faculty of Dentistry, Cairo University, Cairo, Egypt; ^3^Department of Conservative Dentistry, Faculty of Dentistry, Cairo University, Cairo, Egypt; ^4^Oral Biology Department, Faculty of Dentistry, Cairo University, Cairo, Egypt; ^5^Clinic for Conservative Dentistry and Periodontology, School of Dental Medicine, Christian Albrechts University, Kiel, Germany; ^6^Oral Medicine and Periodontology Department, Faculty of Dentistry, Cairo University, Cairo, Egypt

## Abstract

Stem/progenitor cells are undifferentiated cells characterized by their exclusive ability for self-renewal and multilineage differentiation potential. In recent years, researchers and investigations explored the prospect of employing stem/progenitor cell therapy in regenerative medicine, especially stem/progenitor cells originating from the oral tissues. In this context, the regeneration of the lost dental tissues including enamel, dentin, and the dental pulp are pivotal targets for stem/progenitor cell therapy. The present review elaborates on the different sources of stem/progenitor cells and their potential clinical applications to regenerate enamel, dentin, and the dental pulpal tissues.

## 1. Introduction

Dental caries is globally considered among the most prevalent bacterially induced diseases, resulting in enamel and dentin destruction. If untreated, the destruction will mostly lead to irreversible pulpal tissue damage [1]. Currently, the classical line of treatment involves the removal of the affected dental tissues and their subsequent replacement with artificial filling materials, with divergent physical and functional properties [1]. Due to various negative consequences of the restorative techniques and inherent deficiencies of the restoration materials, the ideal solutions to replace defective dental structures could be by biologically restoring/regenerating the lost dental tissues. The development of such new alternative treatment methods is currently considered as an important goal for the dental therapeutic researches.

Mesenchymal stem/progenitor cells (MSCs) are unspecialized plastic-adherent cells with the ability for self-renewal and multilineage differentiation [2] into multiple cell lineages [3]. They have been isolated from a variety of dental tissues, including dental pulp stem cells (DPSCs), stem/progenitor cells isolated from the human pulp of exfoliated deciduous teeth (SHED), periodontal ligament stem/progenitor cells (PDLSCs), stem/progenitor cells from apical papilla (SCAP), alveolar bone-proper-derived stem/progenitor cells (AB-MSCs), gingival mesenchymal stem/progenitor cells (GMSCs), and dental follicle stem/progenitor cells (DFSCs) [4, 5]. The stem/progenitor cells derived from the oral cavity express several mesenchymal markers, including CD29, CD73, CD90, and CD105, as well as embryonic markers such as Sox2, Nanog, and Oct4 [6], but lack the expression of hematopoietic markers, including CD34, CD45, and HLA-DR. Relying on their remarkable proliferative ability and differentiation potential, these stem/progenitor cells are believed to be very promising in the development of future therapeutic approaches to regenerate the enamel, dentin, and pulpal tissues [7].

## 2. The Tissue Engineering Triad

Tissue engineering is an interdisciplinary field that applies the principles of engineering and life sciences towards the development of biological substitutes that could restore, maintain, or improve tissue and organ functions [8]. The concept of tissue engineering relies on the employment of a triad of stem/progenitor cells, scaffolds, and growth factors [8, 9] to regenerate functional biological tissues. Scaffolds have to be implemented with a suitable choice of cells and signaling molecules to initiate the formation of a new dental tissue that can homogenize with the surrounding tissues [10–12].

Numerous stem cell sources have been identified to play an essential role in tissue regeneration. Stem cells are either embryonic or adult stem cells [13]. Embryonic stem cells are immature, undifferentiated cells derived from the inner cell mass of blastocysts [14, 15], with the ability to undergo continuous self-renewal and differentiation. Adult stem/progenitor cells are undifferentiated cells that are capable of differentiating into certain types of tissues [3]. They maintain the integrity of tissues they reside in such as blood, skin, bone, and dental pulp [16].

Scaffolds could be natural polymers (e.g., collagen, chitosan, alginate, and hyaluronic acid) or synthetic materials (e.g., polyglycolic acid, polylactic acid, and polylactic polyglycolic acid) and bioactive ceramics, with each category having its merits as well as limitations in use [17]. Scaffolds could be utilized as a cell support tool, upon which cells are cultured in vitro, prior to their transplantation together with their produced matrix in vivo. Scaffolds can further be employed as growth factor/drug delivery tools, to attract body cells to the scaffold site in vivo for new tissue formation [18]. In this context, scaffolds are essential to structurally support and transport growth factors, DNA, biologically active proteins, and cells as well as provide physical signals important for biological repair/regeneration processes [19, 20]. Aside from these, the topography, architecture, and composition of scaffolds can interact and affect cell response and subsequent tissue formation [18]. It is important for scaffolds to mimic the natural extracellular matrix of the tissue to be replaced [21, 22]. Optimum design for dental tissue regeneration should be made to achieve mechanical integrity and functionality and to help in cell adhesion and differentiation.

As a third important factor in the tissue engineering triad, growth factors were suggested to be crucial for the regenerative process. They are normally released from cells and are directly presented to cell surface receptors through their interaction with the neighboring extracellular matrix. Binding of growth factors to particular cell-membrane-linked receptors activates various mechanisms and pathways involved in tissue engineering such as cell migration, survival, adhesion, proliferation, growth, and differentiation into the desired cell type [23–27]. Especially, bone morphogenetic protein- (BMP-) 2 was shown to induce the differentiation of dental pulp stem/progenitor cells into odontoblasts [23]. It was also demonstrated that BMP-4 mediates the differentiation of human embryonic stem cells into dental epithelium with the ability for tooth formation [28]. In addition, transforming growth factor-*β* (TGF-*β*) promotes the differentiation of odontoblast-like cells and stimulates dental pulp stem cell-mediated mineralization [23]. Broadly speaking, these growth factor-mediated cell responses are crucial for growth, wound healing, and angiogenesis in repair/regeneration processes [26].

## 3. Enamel Regeneration

### 3.1. Enamel Structure and Amelogenesis

Enamel, the hardest tissue in the human body, is a highly organized dental tissue, covering the outer layer of the tooth crown. It possesses unique mechanical and structural properties [29–32], relying on its high hydroxyapatite content, the arrangement of apatite crystals into enamel prisms, and finally, the alignment of these prisms in a picket-fence appearance in a tissue of high physical resilience and great hardness [33–37]. Ameloblasts, the enamel-forming cells, are specialized epithelial cells differentiating from the inner cells of the enamel organ [38]. They exhibit polarization and elongation with a pronounced Golgi apparatus and endoplasmic reticulum to form and secrete enamel proteins and influx phosphate and calcium ions into the forming enamel matrix [39, 40]. Enamel proteins are necessary for enamel formation, with amelogenin, ameloblastin, and enamelin being the three major proteins observed in the developing teeth [41]. Recently, this list was amended by amelotin and odontogenic ameloblast-associated protein (ODAM), which were observed in the junctional epithelium and during the maturation stage of amelogenesis [42–45]. Once the enamel matrix is formed, ameloblasts reabsorb water and degrade enamel proteins during the maturation stage of amelogenesis [39, 40]. Finally, they undergo apoptosis and the mature enamel becomes acellular. As a result, once damaged, unlike other biomineralized hard tissues such as dentin and bone, the enamel cannot regenerate by itself [37, 46]. Therefore, a reparative healing of destroyed enamel depends mainly, if at all, on acellular remineralization of superficial demineralized defects [47].

To restore enamel defects either due to caries, trauma, or others, artificial materials were manufactured to resemble its hardness [48]. Unfortunately, most of the current materials do not possess the same mechanical, physical, and esthetic properties of the lost tissues [49]. Despite the urgent need for tooth enamel regeneration, enamel tissue engineering is facing many difficulties [50–53], including the complex posttranslational protein modifications required for crystal growth [54] and the recapitulation of the unique movements of ameloblasts during the organization of hydroxyapatite crystals into the enamel prisms [55]. Despite all these trials and findings, to date, there exists no scheme for viable cell-based in vivo enamel tissue engineering [37]. The main challenge remains to produce an artificial enamel that resembles the prismatic and interprismatic patterns of natural enamel, has the proper anatomy, and can substitute for lost enamel-forming cells [56].

### 3.2. Cells

As enamel-forming cells are lost following tooth development, alternative cellular sources were needed to bring about a cellular-based regeneration. In this context, nondental epithelium-derived human cells, including gingival epithelial cells [57], induced pluripotent stem cells (iPSCs) [58], and human keratinocyte stem cells (hKSCs) [59, 60] were suggested to differentiate into enamel-forming ameloblasts when combined with mouse or human embryonic dental mesenchyme. Still, only a small percentage of these explants in subrenal cultures demonstrated the formation of dental enamel [60]. Embryonic stem/progenitor cells were similarly demonstrated to differentiate into oral ectoderm and dental epithelium, using variable concentrations of BMP-4 [61]. The formed dental epithelium, when mixed with mouse embryonic dental mesenchyme and transplanted into renal capsules for thirty days, subsequently generated teeth-like structures, including dentin and enamel, with an incisor-like appearance [28]. Similarly, human keratinocyte stem cells when combined with embryonic mouse dental mesenchyme, sonic hedgehog (SHH), and fibroblast growth factor 8- (Fgf8-) soaked agarose beads as reconstructed tooth germs [62] and transplanted into mice renal capsules, demonstrated ameloblastic differentiation with enamel deposition. Mouse induced pluripotent stem cells demonstrated a differentiation into ameloblast-like cells, using epithelial rests of Malassez cell-conditioned medium and gelatin-coated dishes, with high expression of amelogenin, ameloblastin, and keratin 14 [63].

Similarly, the Hertwig epithelial root sheath (HERS) and epithelial rests of Malassez (ERM) cells demonstrate a remarkable ability to produce enamel matrix proteins [64]. HERS cells entrapped in cementum produced amelogenin, ameloblastin, amelotin, and ODAM [45]. It was found that primary cultured HERS/ERM cells possess a primitive stem/progenitor cell population that exhibits embryonic stem cell and epithelial markers [57, 65]. Ex vivo-expanded ERM exhibited both bone marrow mesenchymal stem/progenitor cell- (heat shock protein-90b, CD44 and CD29) and epithelial cell-markers (epithelial membrane protein-1, cytokeratin-8, and E-cadherin) proving their stem/progenitor cell-like properties [66]. An ERM cell line was further successfully generated from human periodontium to be used for future research [67]. ERM cells when cocultured with dental pulp cells were differentiated into ameloblast-like cells and produced enamel-like tissues [68]. Immortalized odontogenic epithelial cells isolated from ERM expressed stem-cell-related genes and generated calcification foci when transplanted into immunocompromised mice [69].

Odontogenic epithelial stem cells (OEpSCs) were first observed in the continuously growing rodent incisors. They are of epithelial origin, interact reciprocally with the mesenchymal stem/progenitor cells of ectomesenchymal origin [70], and possess the ability to generate all the epithelial tissues of the tooth, including the enamel-forming ameloblastic layer [71–73]. In postnatal life, OEpSCs were identified in the epithelial rests of Malassez (ERM) usually present near the incomplete root ends; the junctional epithelium (JE) which surrounds the neck of teeth; the reduced enamel epithelium (REE) covering the newly erupting tooth; the dental lamina (DL) and its remnants, known as cell rests of Serres, in the retromolar area; and the remnants of DL in the gubernaculum cord (GC), found above any erupting tooth [74]. Various genes were recognized in OEpSCs, including Bmi, Sox2, Yap, ABCG2, Lgr5, Oct3/4, Gli1, and Lrig1 [72, 73, 75–78]. It was demonstrated that Sox2+ odontogenic epithelial stem cells are able to produce teeth. The odontogenic epithelial stem cell niche was proved to be regulated by Fgf10 [79, 80].

### 3.3. Scaffolds and Biodegradable Materials

To successfully culture a patterned enamel organ, it was found that a proper three-dimensional scaffold such as a collagen sponge in combination with feeder cells such as NIH 3T3 mouse fibroblast cells should be present [81–83] to support and compensate for the epithelial-mesenchymal interactions that occur during early tooth formation [84]. Collagen sponge scaffolds and gels were demonstrated to help in cell attachment, proliferation, and differentiation as well as in the formation of calcified tissues [85]. Primary enamel organ cells cultured on feeder cells expressed many enamel proteins as kallikrein 4, ameloblastin, amelogenin, and matrix metalloproteinase (MMP) 20 [83]. Enamel organ epithelial (EOE) cells combined with dental pulp cells in scaffolds produced enamel with amelogenin expression in tall columnar epithelial cells found on enamel and dentin surfaces [48]. A three-dimensional multilayered macroscale biomimetic coculture system, using chitosan and type I collagen was similarly seeded with mesenchymal-derived dental pulp stem cells and HAT-7 dental epithelial cells to simulate epithelial-mesenchymal interactions. This system enabled the coculture of epithelial and mesenchymal cells, and the movement of the two cell types in various directions and Ca deposits were observed [86].

Still, available information is very limited and greatly diverse to distinguish the characteristics of each specific scaffold and its impact on possible stem/progenitor cell-mediated enamel regenerative outcomes [87].

### 3.4. Signaling Molecules in Amelogenesis

**Figure 1:**
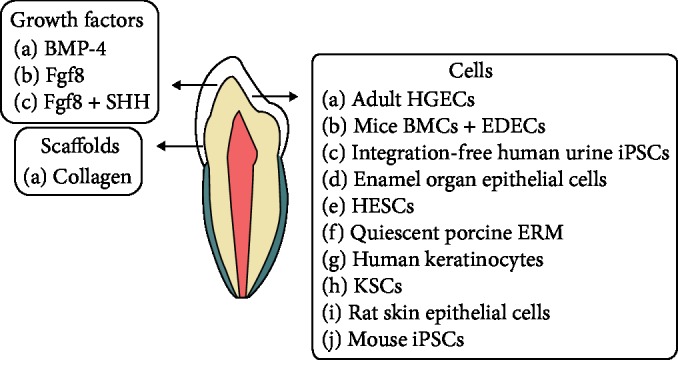
Diagram showing cells, growth factors, and scaffolds examined in the field of enamel regeneration.

Various signaling molecules were proposed to be involved in the epithelial-mesenchymal interactions that occur during odontogenesis, including fibroblast growth factor (Fgf), sonic hedgehog (SHH), wingless (Wnt), bone morphogenic protein (BMP), and transforming growth factor *β* (TGF-*β*) [88, 89]. Activin, BMP, and Fgf signals in the epithelial stem cell niche regulate enamel deposition in mice incisors [90]. Mesenchymal signals involved in ameloblast induction include TGF-*β*1, BMP-4, and BMP-2 [91, 92]. SHH signaling preserves the stem cell niche present in the molar cervical loops [93]. FAK-YAP-mTOR signaling maintains the equilibrium between stem cell proliferation and differentiation towards ameloblast lineage [94]. BMP signaling was demonstrated to be crucial for ameloblast differentiation [91] and enamel formation [95]. Ectodysplasin (Eda), a signal found in primary and secondary enamel knots and the placodes of all ectodermal appendages, is considered a key regulator of ectodermal organ development, including molecules from vital signaling pathways such as SHH and Fgf20 [96]. In addition, SHH found in epithelial stratum intermedium cells support ameloblastic differentiation and maturation [97, 98]. Other epithelial signaling molecules that regulate ameloblasts are Wnt3, TGF-*β*1, Follistatin, and Eda. Ameloblasts also express transcription factors such as Msx2 and Sp6 that have important roles in amelogenesis [99]. Regulating these molecules could help to generate ameloblast lineage precursors resembling odontogenic epithelial stem/progenitor cells that could be utilized in enamel regeneration approaches [6] (Table 1 and Figure 1).

## 4. Dentin Regeneration

### 4.1. Dentin Structure and Dentinogenesis

The pulp-dentin complex originates embryonically from the neural crest ectomesenchyme [100]. Odontoblasts are differentiated at the late bell stage of tooth development, and their major function is to secrete the extracellular dentin matrix components (ECM), followed by their mineralization, generating the primary dentin, the main bulk of the circumpulpal dentin matrix, and completing root formation. Secondary dentin is laid down as a physiological process throughout life, while tertiary dentin is generated at the pulp-dentin interface in response to environmental stimuli. Tertiary dentin might be reactionary (structurally similar to physiological dentin) or reparative (poorly organized, mainly atubular structure, with cells trapped within the matrix). Each type arises from two different populations of cells, original postmitotic odontoblasts and newly generated cells derived from the pulp (dental pulp stem/progenitor cells (DPSCs)), respectively [101, 102].

### 4.2. Cells

Aside from the attempts for enamel regeneration, stem/progenitor cell-based tissue engineering remains a promising modality for functional dentin regeneration [103–105]. A lineage-tracing study proved that new odontoblasts generated during reparative dentinogenesis in teeth come up from the perivascular cells identified by *α*-smooth muscle actin (*α*SMA) expression. Furthermore, it was demonstrated that the progeny of the *α*SMA+ population scarcely participated in physiological dentin deposition [106]. Coimplantation of MSCs and ECs accelerated pulp healing with a complete dentin bridge formation [107]. Swine autologous dental pulp stem/progenitor cells (sDPSCs) transferred via hydrogel and transplanted into a mini swine root model showed that vascularized pulp-like tissue and a layer of newly deposited dentin (reparative dentin) were deposited along the canal walls with the creation of a dentin bridge-like structure [108]. A further in vivo study showed that iPSCs generated a pulp-like tissue with functional odontoblasts capable of producing tubular dentin-like structures [109]. In vitro investigation demonstrated that utilizing SHED in combination with different pulp capping materials stimulated proliferation, migration, and odontogenic-like phenotype differentiation of the cells [110].

### 4.3. Scaffolds and Biodegradable Materials

Several successful in vitro studies tested variable biomaterials to promote dentin regeneration. A biomembrane composed of a chitosan/collagen matrix embedded with calcium-aluminate microparticles proved to induce the differentiation of HDPCs into odontoblast-like cells, with the deposition of a significant amount of mineralized matrix [111]. Culturing of DPSCs onto human-treated dentin (hTD) regenerated dentin-like tissues [112, 113]. Similarly, fibrin proved to enhance pulp-like tissue generation as well as odontoblast differentiation, with dentin sialoprotein expression [114].

An attempt to utilize a biodegradable collagen sponge as a delivery vehicle for molecules like MTA or other experimental small-molecule GSK3 inhibitors promoted tertiary dentin formation in deep dental lesions, following experimentally induced pulp exposure [115]. Ceramic scaffolds, such as calcium phosphates (Ca/P) and bioactive glasses or glass ceramics, were further tested. Ca/P scaffolds contain tricalcium phosphate (TCP) or hydroxyapatite (HA), which are notably related to the mineralization of the matrix of the tooth [116]. Calcium hydroxide, Mineral Trioxide Aggregate (MTA), and Biodentine were reported to aid in the formation of the tertiary dentin [117]. Another in vitro study tested three capping materials, namely, mineral trioxide aggregate (MTA), calcium hydroxide (CH), and Biodentine (BD), and proved that these materials are biocompatible and could stimulate proliferation, migration, and differentiation of SHED [110]. Nanofibrous spongy microspheres (NF-SMS), nanofibrous microspheres (NF-MS) without a pore structure, and conventional solid microspheres (S-MS) with neither nanofibers nor pore structure were further tested for dentin regeneration. The biodegradable and biocompatible poly(L-lactic acid) block-poly(L-lysine) were fabricated into the NF-SMS with interconnected pores, enhancing the proliferation and odontogenic differentiation of HDPSCs. NF-SMS provided superior dentin-like tissue formation compared to NF-MS or S-MS with a remarkable level of mineralization [118].

The application of biological printing combined with dental stem/progenitor cells employing clinical methods of 3D biofabrication and regeneration of dental tissues are the currently suggested alternative to classical dental restorations. The use of bioinks enabled the synthesis of scaffolds with precise, reproducible microarchitectures. Novel dentin-derived extracellular matrix (ECM) hybrid cell-laden hydrogel bioinks, synthesized from alginate and dentin matrix proteins were characterized and showed high printability and cell survival at different concentrations. Moreover, these hybrid hydrogels demonstrated the ability to be embedded with acid-soluble dentin molecules, enhancing odontogenic differentiation of SCAPs and effectively engineering the pulp-dentin complex [119].

### 4.4. Signaling Molecules

As previously mentioned, BMP-2 controls odontoblastic differentiation of dental pulp stem cells and transforming growth factor-*β* (TGF-*β*) can stimulate odontoblast-like cell differentiation and DPSC-mediated mineralization [23]. Also, platelet-derived growth factor (PDGFBB) and dentin-derived growth factors (eDMP) proved to enhance HDPSC proliferation and odontoblastic differentiation, generating dentin-like mineralized tissues [120, 121]. G-CSF enhanced the proliferation and migration activity of stem/progenitor cells with dentin regeneration [122]. It was reported that the histone demethylation enzyme, lysine demethylase 1A (KDM1A), can regulate the directed differentiation in odontogenic MSCs by forming KDM1A and PLOD2 (procollagene-lysine2, oxoglutarate5-dioxygenase2) protein complex. It was reported that KDM1A in SCAP regulatory mechanisms of dynamic osteo/dentinogenic differentiation showed more diverse outcomes when applied in vitro than in vivo. However, in the final outcome of KDM1A inhibition, it promoted osteo/dentinogenesis in vivo [123]. Moreover, H2S proved to aid in the differentiation of DPSCs and dentin formation in vitro and in vivo via Ca^2+^ homeostasis and Ca^2+^ influx/GSK3*β*/(glycogen synthase kinase-3*β*) *β*-catenin cascade response. Also, it was evident that *β*-catenin signaling plays crucial roles in dentin formation [124].

**Figure 2:**
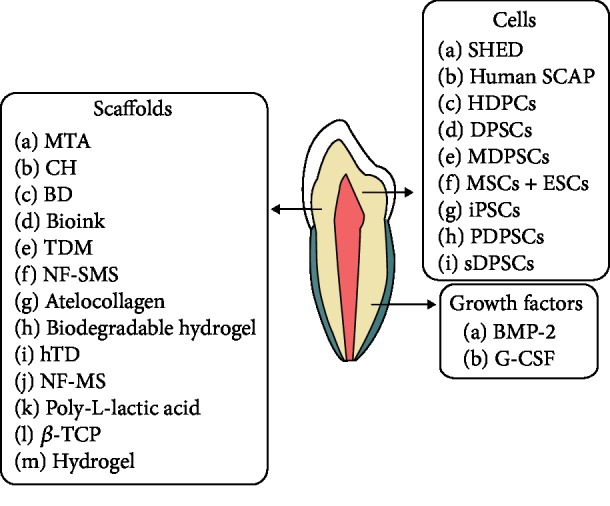
Diagram showing cells, growth factors, and scaffolds examined in the field of dentin regeneration.

Simvastatin (SIM), a drug commonly used to treat hyperlipidemia, was further reported to enhance odontogenic differentiation and accelerate mineralized tissue formation and de novo dentin formation [125, 126]. Combining SIM with canine DPSCs enhanced coronal pulp regeneration as well as dentin regeneration effectively and rapidly in beagle dogs. Small molecule inhibitors of glycogen synthase kinase 3 (GSK3) used in clinical trials for the treatment of neurological disorders such as Alzheimer's disease stimulated reparative dentine formation, with naturally generated new dentine at sites of damage [115, 127]. Smaphorin 3A (Sema 3A) and its receptor Nrp1, usually expressed in rat dental pulp tissue and human DPSCs, were thought to be potent factors capable of inducing differentiation of DPSCs into odontoblasts. Sema3A application to dental pulp exposure sites in a rat model induced effective reparative dentin reconstruction and promoted the formation of an odontoblastic layer, dental tubules, and predentin [128] (Table 2 and Figure 2).

## 5. Pulpal Tissue Regeneration

### 5.1. Pulpal Tissue Structure

Dental pulp is the soft tissue located in the center of the tooth, and it is surrounded by dentin. The primary function of the pulp is formative; it gives rise to odontoblasts that form dentin. Odontoblasts are the most distinctive cells of the pulp. They form a single layer at the periphery and synthesize the matrix, which becomes mineralized and form dentin. As previously discussed, the pulp-dentin complex originates embryonically from the neural crest ectomesenchyme and constitutes physiologically and functionally a single unit, providing vital functions for tooth homeostasis [100].

The dental pulp is a richly vascularized and innervated connective tissue comprising heterogeneous cell populations, among which stem/progenitor cells are anticipated to constantly replenish odontoblasts to form secondary and tertiary/reparative dentin throughout adult life [101, 102, 129, 130]. Mesenchymal stem/progenitor cell transplantation into endodontically treated root canals was attempted to regenerate the damaged dental pulp-dentin complex [131]. Although most of the research conducted on stem/progenitor cell-mediated reparative/regenerative endodontics used animal models, initial human clinical data are available now.

### 5.2. Cells

Coimplementation of endothelial cells with MSCs induced the acceleration of pulp tissue regeneration/healing and dentin bridge formation together with the upregulation of proangiogenic factors and the formation of a more organized dental pulp-like tissue and a thicker dentin bridge [107, 131–133]. Porcine deciduous pulp stem/progenitor cells (PDPSCs) transplanted to repair pulp chamber roof defects in the premolars of swine showed that after 16 weeks they regenerated dentin-like structures and nearly completely restored pulp chamber roof defects [134]. MDPSC (mobilized dental pulp stem cell) transplantation into pulpectomized teeth with G-CSF resulted in pulp/dentin regeneration as was evident by electric pulp testing, magnetic resonance imaging, and cone beam computed tomography [135]. Transplanted autogenous HDPSCs (human dental pulp stem cells), regenerated, innervated, and vascularized dental pulpal tissue in 26 patients with root length completion and apical foramen closure. Following up patients with the implanted HDPSCs for 24 months did not demonstrate any adverse events [136]. DPSCs (dental pulp stem cells) isolated from an inflamed third molar after being extracted and cultured then inoculated in another tooth of the same patient showed a normal periapical area after 3-year follow-up using cone beam computed tomography [137]. Hence stem/progenitor cell transplantation holds a promising potential for dentin/pulp complex regeneration.

### 5.3. Scaffolds and Biodegradable Materials

Scaffolds harboring the appropriate growth/differentiation factors are very important for the success of pulpal tissue regeneration [132]. These scaffolds should mimic the natural pulpal microenvironment, providing the necessary structural signals, adhesion molecules, and pore sizes for homing, differentiation, and cellular phenotypic conversion, through permitting cell-matrix and cell-cell interactions [138]. Different scaffolds were used in different studies such as mineralized *β*-tricalcium phosphate carrier/scaffolds [134], injectable collagen [122, 139, 140] and hydrogel-chitosan carriers [141], and gelatin sponge [126]. Platelet-rich fibrin (PRF), centrifuged from the patient's own blood samples, was suggested as a natural scaffold for pulpal tissue regeneration. PRF introduced inside the root canal allows cellular migration, cytokine enmeshment, and slow continuous release of cytokines such as platelet-derived growth factor, transforming growth factor beta 1, fibroblast growth factor, and vascular endothelial growth factor from 7 to 28 days, achieving the peak level on day 14 [142]. In addition, it provides a strong firm architecture and a specific 3-dimensional distribution of platelets and leukocytes [137]. A novel transplant consisting of cell-sheet fragments of DPSCs and PRF granules proved to regenerate pulp-dentin-like tissues in the root canal, both subcutaneously in nude mice and in the roots of canines. It induced a favorable regeneration of compact pulp-like tissues, and a remarkable deposition of regenerated dentin along the intracanal walls at 8 weeks postoperation was observed [143]. Still, the impact of the characteristics of carrier/scaffolds on the transplanted stem/progenitor cell-mediated regenerative outcomes are currently only partly elucidated [87].

### 5.4. Signaling Molecules

**Figure 3:**
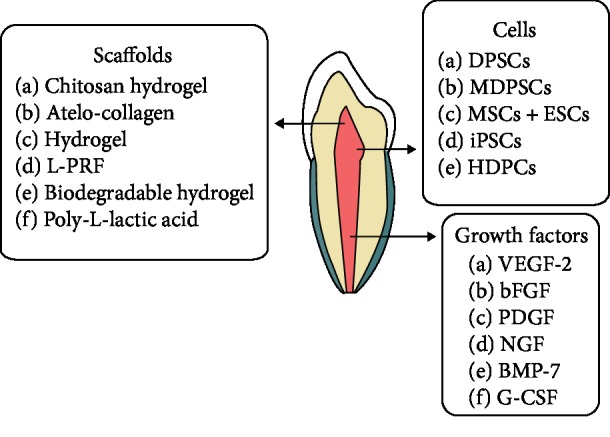
Diagram showing cells, growth factors, and scaffolds examined in the field of pulp regeneration.

Similar to enamel and dentin formation, cytokines or signaling molecules participate in pulp regeneration through their ability to mobilize endogenous cells and to regulate the proliferation and differentiation of the stem/progenitor cells [144]. Signaling molecules have been used and added to the scaffolds for proliferation, differentiation, and survival of stem/progenitor cells, with potentially important roles in signaling during pulp regeneration. Several studies suggested that many cytokines and growth factors were involved in promoting chemotaxis, proliferation, and differentiation of the stem/progenitor cells inside the root canal which led to generation of new tissues [145–147]. Transplantation of processed autologous dental pulp with growth factors (vascular endothelial growth factor-2 (VEGF-2), basic fibroblast growth factor (bFGF), platelet-derived growth factor (PDGF), nerve growth factor (NGF), and bone morphogenetic protein-7 (BMP-7)) embedded in a chitosan hydrogel scaffold was useful in regenerating pulp and dentin-like tissues in necrotic immature permanent teeth with apical periodontitis in dogs [141] with promising results. G-CSF in combination with DPSCs demonstrated pulpal tissue regeneration, vascularization, and nerve regeneration [148]. Basic fibroblast growth factor (bFGF) was demonstrated to be a potent homing/migration factor in pulp regeneration therapy similar to the influence of G-CSF [138]. Mobilized dental pulp stem cells and granulocyte colony-stimulating factor (G-CSF) with collagen transplanted into mature pulpectomized dogs' teeth completely filled the root canal with pulp-like tissue with large blood vessels and secondary dentin formation. However, MRI examination implied that the regenerated dentin might be undermineralized [149]. Another study revealed that stem cell factor (SCF) can accelerate cell homing and the maturation of the pulp-dentin complex in human immature teeth, as well as proliferation and odonto/osteogenic differentiation [150]. Still, the ideal constellation of growth/differentiation factors for functional pulpal regeneration remains largely unknown (Table 2 and Figure 3).

## 6. Conclusion

Stem/progenitor cell-based tissue engineering and bioprinting are promising approaches to protect the vitality and restore the integrity of dental tissues. Many attempts proved to be very promising, as reported in various in vitro studies, animal studies, and very few human trials. Despite the fact that the proposed biomaterials and techniques could be promising for future dental tissues' regeneration, still the complexity and the multicellular interactions naturally existing in dental structures represent great currently unsolved challenges. A clear set of universally accepted markers for the isolation and characterization of stem/progenitor cells and the development of serum and animal product-free culturing media for cell expansion are further major hurdles prior to considering stem/progenitor cell-based transplantation therapies for routine clinical application. Finally, the side effects of stem/progenitor transplantation should be clearly investigated, prior to becoming a clinical therapeutic reality in restorative dentistry.

## Figures and Tables

**Table 1:** Summary of studies on enamel regeneration.

Enamel regeneration				
	Cells	Carrier/scaffold	Growth factors	Outcome
Angelova et al. 2013 [57]	Adult human gingival epithelial cells	—	—	Tooth-like structures Presence of enamel spaces and ameloblast-like cell populations

Hu et al. 2006	Mice bone marrow cells+mouse embryonic dental epithelial cells	—	—	Formation of nondividing, polarized, and secretory ameloblast-like cells without cell fusion

Cai et al. 2013 [58]	Integration-free human urine induced pluripotent stem cells	—	—	Tooth-like structures having elastic modulus and hardness similar to human tooth and containing enamel space and enamel organ Presence of ameloblasts with a ruffled border-like structure and papillary layer Expression of ameloblastin

Honda et al. 2005 [48]	Enamel organ epithelial cells	—	—	Production of enamel Expression of amelogenin in tall columnar epithelial cells found on enamel surface

Li et al. 2019 [28]	Human embryonic stem cells	—	Bone morphogenetic protein-4 (BMP-4)	Teeth-like structures Newly generated tooth-like structures contained enamel spaces similar to natural teeth

Shinmura et al. 2008 [68]	Quiescent porcine epithelial cell rests of Malassez from PDL of deciduous incisor teeth	Collagen sponge	—	Enamel-like tissues Positive staining for amelogenin in the enamel-like tissues Presence of well-developed ameloblasts

Wang et al. 2010 [60]	Human keratinocytes	—	Fibroblast growth factor 8 (Fgf8)	Epithelial cells became elongated and deposited enamel Immunohistochemical assays demonstrated the presence of ameloblastin and MMP-20

Hu et al. 2018 [62]	Human keratinocyte stem cells	—	Fibroblast growth factor 8 and Sonic hedgehog (Fgf8+SHH)	Tooth-like structures Intact prisms of the regenerated enamel

Liu et al. 2013	Rat skin epithelial cells	—	—	Enamel-dentin-like tooth germ-like structures

Yoshida et al. 2015 [63]	Mouse induced pluripotent stem cells (Mouse iPS)	—	—	Ameloblast-like cells Expression of high levels of amelogenin and ameloblastin

**Table 2:** Summary of studies on dentin/pulp regeneration.

Dentin/pulp regeneration				
	Cells	Carrier/scaffold	Growth factors	Outcome
Araujo et al. 2018 [110]	Stem cells from human exfoliated deciduous teeth (SHED)	Mineral trioxide aggregate (MTA) Calcium hydroxide (CH) Biodentine (BD)		The three tested materials maintained viability and stimulated proliferation, migration, and odontogenic-like phenotype differentiation

Athirasala et al. 2018 [119]	Human stem cells of apical papilla (human SCAP)	Bioink: printable alginate hydrogels with the soluble and insoluble fractions of dentin matrix		Odontogenic differentiation of SCAPs

Chen et al. 2017	Human dental pulp cells (HDPCs)	Human and porcine treated dentin matrix (TDM)		Complete dentin bridge formation Regeneration of reactionary dentin

El Ashiry 2018 [141]	Dental pulp stem cells (DPSCs)	Chitosan hydrogel scaffold	Vascular endothelial growth factor (VEGF-2) Basic fibroblast growth factor (bFGF) Platelet-derived growth factor (PDGF) Nerve growth factor (NGF) Bone morphogenetic protein-7 (BMP-7).	Periapical radiolucency healing Radicular lengthening Radicular thickening Apical closure

Iohara 2013 [148]	Dental pulp stem cells (DPSCs) Allogenic	Atelocollagen; collagen	G-CSF (granulocyte colony-stimulating factor)	Vascularization and neural regeneration in the DPSC group

Jia et al. 2016 [126]	Dental pulp stem cells (DPSCs)	—	—	Simvastatin stimulates DPSC-induced pulp and dentin regeneration after pulpotomy.

Kuang et al. 2015 [118]	Human dental pulp cells (HDPCs)	Nanofibrous spongy microspheres (NF-SMS)		Dentin-like tissue formation

Mangione et al. 2017	Dental pulp stem cells (DPSCs)	Hydrogel		Failure of partial pulp regeneration

Meza et al. 2019 [137]	Dental pulp stem cells (DPSCs) Autologous	Leukocyte-platelet-rich fibrin (L-PRF)		Six-month and 3-year follow-ups Periapical index (PAI) score of 1 Cone beam computed tomographic periapical index (CBCT PAI) score of 0

Nakashima 2017	Isolated human mobilized dental pulp stem cells (MDPSCs) Autologous	Atelocollagen scaffold	G-CSF (granulocyte colony-stimulating factor)	*CBCT evaluation*: Continued thickening of radicular walls (lateral dentin formation) Decrease in the volume of the dental pulp between 16 and 28 weeks *MRI evaluation of apical closure*: Relative signal intensity of apical part of root canal at 24 weeks *EPT (electric pulp tester) for evaluation of the sensibility of teeth*: Positive response in 4 cases out of the 5 cases

Sueyama et al. 2017 [107]	MSCs (mesenchymal stem cells) with ECs (endothelial stem cells)	Biodegradable hydrogel-made scaffolds		Pulp tissue regeneration/healing with complete dentin bridge formation

Tran et al. 2015 [112]	HDPCs	Human-treated dentin (hTD)		Regeneration of dentin-like tissues and expression of specific dentin markers

Wang et al. 2016 [105]	Human SCAP	NF-MS nanofibrous microspheres	Bone morphogenetic protein-2 (BMP-2)	Newly synthesized matrix and dentin-like tissues were present in BMP-2-treated groups

Xie et al. 2017	Induced pluripotent stem cells (iPSCs)	Poly-L-lactic acid (Boehringer Ingelheim) scaffold cast		Production of pulp-like tissue with functional odontoblasts capable of generating tubular dentin-like structures in vivo

Xuan et al. 2018 [136]	HDPSCs (human dental pulp stem cells) Autologous	—	—	Continued root lengthening and apical closure Increase in vascular formation At 12 months after treatment, laser Doppler flowmetry showed a mean increase in vascular formation

Zheng et al. 2012 [134]	Porcine deciduous pulp stem/progenitor cells (PDPSCs)	beta-Tricalcium phosphate (*β*-TCP)		Dentin-like structure Completely restored pulp chamber roof defects

Zu et al. 2018	Autologous swine dental pulp stem cells (sDPSCs)	Hydrogel		Dentin bridge formation

## Abbreviations

BMP-4/2/7: Bone morphogenic protein-4/2/7
Fgf8: Fibroblast growth factor-8
SHH: Sonic hedgehog
HGECs: Human gingival epithelial cells
BMCs: Bone marrow cells
EDECs: Embryonic dental epithelial cells
iPSCs: Induced pluripotent stem cells
HESCs: Human embryonic stem cells
ERM: Epithelial rests of Malassez
KSCs: Keratinocyte stem cells
MTA: Mineral trioxide aggregate
CH: Calcium hydroxide
BD: Biodentine
TDM: Treated dentin matrix
NF-SMS: Nanofibrous spongy microspheres
hTD: Human-treated dentin
NF-MS: Nanofibrous microspheres
*β*-TCP: *β*-Tricalcium phosphate
SHED: Stem cells from human exfoliated deciduous teeth
SCAP: Stem cells from apical papilla
HDPSCs: Human dental pulp stem cells
DPSCs: Dental pulp stem cells
MDPSCs: Mobilized dental pulp stem cells
MSCs: Mesenchymal stem cells
ESCs: Endothelial stem cells
PDPSCs: Pig dental pulp stem cells
sDPSCs: Swine dental pulp stem cells
G-CSF: Granulocyte colony-stimulating factor
L-PRF: Leucocyte-platelet-rich fibrin
VEGF-2: Vascular endothelial growth factor-2
bFGF: Basic fibroblast growth factor
PDGF: Platelet-derived growth factor
NGF: Nerve growth factor.
